
JOURNAL INFORMATION
==============================
NLM Title Abbreviation: J Child Orthop
Journal ID: J Child Orthop
NLM Journal ID: 101313582

Journal of Children's Orthopaedics

ISSN: 1863-2521
EISSN: 1863-2548
Publisher: SAGE Publications

ARTICLE INFORMATION
==============================
PMCID: PMC4353812
PMID: 25711755
DOI: 10.1007/s11832-015-0641-4
Article ID: 641
Article version: 1
Subjects: Abstracts

IFPOS 34th Annual Meeting

Electronic publication date: 2015 Feb 25
Print publication date: 2015 Apr
Volume: 9
Issue: Suppl 1
First page: 133
Last page: 138
Copyright: © The Author(s) 2015
License: This article is distributed under the terms of the Creative Commons Attribution License which permits any use, distribution, and reproduction in any medium, provided the original author(s) and the source are credited.
License URL: https://creativecommons.org/licenses/by/4.0/

issue-copyright-statement: © The Author(s) 2015

==============================

## IEP1

### External fixation of pediatric subtrochanteric fractures using calcar rather than neck pins

**Sherif Galal**[1]

[1]Cairo University, Cairo, Egypt

**Trauma/Lower extremity**

*Objective:* Subtrochanteric femoral fractures in children are uncommon and have received limited attention in the literature. Its treatment is controversial, different options are available: traction, spica casting, internal fixation and external fixation. The aim of this study is to present our results with external fixation of pediatric subtrochanteric femoral fractures using calcar rather than neck pins.
*Design:* Between January 2012 and January 2014 a prospective study of this technique was done at Cairo University Hospital (Level 1 trauma center).
*Patients:* 14 patients with closed subtrochanteric femoral fractures. Average age was 6.4 years (range 3.8–10.2 years). Pathological fractures and fractures associated with neuromuscular diseases were excluded. Two patients were multiply injured.
*Intervention:* An Ilizarov frame was used with 2 proximal half pins inserted from greater to lesser trochanters parallel to the hip joint orientation line and secured to an arch, another 3 half pins were inserted distally perpendicular to the femoral shaft and secured to an arch that was connected by three rods to the proximal arch. No postoperative spica was used. Average follow-up was 18 months (range 12–36 months).
*Outcome:* All fractures united with anatomical alignment within an average of 8 weeks (range 6–12 weeks). There were no deep infections and no significant limb length discrepancies. At the latest follow-up, no patient had any restriction of activities.
*Conclusion:* External fixation with calcar rather than neck pins appears as a good treatment option for subtrochanteric femoral fractures in children.

## IEP2

### A longitudinal survey of factors associated with foot arch development in children

**Chia Chang**[1], Liang Chang[2]

[1]Chang Gung Memorial Hospital, Taoyuan, Taiwan.
[2]Biomedical engineering institu, Taipei, Taiwan

**Hip/Lower extremity/foot and ankle**

*Background:* Children develop foot arch naturally and some have persistent flatfeet to adulthood. A clinical issue is who will develop foot arch soon and who are at risk of persistent flatfeet. This study longitudinally surveyed 6-year-old flatfoot children to determine the factors associated with foot arch development in 1.5 years.
*Methods:* 662 1st grade children had body anthropometric data, physical fitness tests, joint hypermobility scale, and footprints studies at age of 6.7 and 8.3 years. Flatfoot was diagnosed by the trough value of Chippaux-Smirak index (CSI) that showed bimodal frequency distribution. Body and function factors were compared between children developing from flatfoot to non-flatfoot and children remaining in flatfoot by independent t test and Chi-square test.
*Results:* 617 children (93 %) completed two evaluations 1.5 years apart. A trough value of CSI 0.58 determined 294 children in flatfoot status, and they gained more body weight than children with non-flatfoot in subsequent 1.5 years (5.5 vs. 5.1 Kgs, $p = 0.02$). In 294 flatfoot children, 77 (26 %) developed to non-flatfoot and the other 217 children remained in flatfoot in the second survey. Significant differences between children with and without foot arch development were initial CSI value (0.72 vs. 0.75, $p = 0.002$) and change of one leg balance ability (increased by 3.4 vs. 2.0 s, $p = 0.03$).
*Discussion:* This longitudinal study did not support sex or obesity was factor for foot arch development, but flatfoot children gained more weight than non-flatfoot children. Foot arch development coincides with improvement in one leg balance ability. We speculate a subtle motor development that associates better balance skills with foot arch development together.

## IEP3

### Femoral deformities in spastic hip displacement

**Chia Chang**[1], Ken Kuo[2], Shih-Hsun Shen[3], Hsuan-Kai Kao[1], Wei-Chun Lee[1]

[1]Chang Gung Memorial Hospital, Taoyuan, Taiwan.
[2]National Taiwan University Hos, Taipei, Taiwan.
[3]Chang Gung Memorial Hospital, Chiayi, Taiwan

**Cerebral Palsy**

*Background:* Coxa valga and femoral anteversion are regarded as contributing factors for hip displacement in cerebral palsy children. The study is to clarify the relationship between femoral deformities, posture, and hip displacement and to explore the effect of windblown posture on femoral deformities.
*Methods:* We studied32 consecutive non-ambulatory cerebral palsy children (mean age: 8.5 years) who had three-dimensional computed tomography (3D-CT) before surgery for hip displacement. Among them, 24 had windblown posture and 8 had bilateral hip displacement with symmetrical hip motion. Femoral anteversion (FA) and true neck shaft angle (NSA) was measured on reconstructed and realigned 3D-CT images. Hip motion, migration percentage (MP), FA, and NSA from 24 abducted hips (Group A), 24 adducted hips (Group B) from children with windblown posture and 16 displaced hips (Group C) from children without windblown posture were compared by analysis of variance (ANOVA) with Scheffe's post hoc analysis.
*Results:* ANOVA revealed that hip abduction motion, MP, and FA were significantly different among the three groups, but NSA and flexion contracture were not. FA 38.3° in Group A hips was comparable to FA 35.5° in Group C hips, though MP was significantly different between the two groups. FA 45.0° in Group B hips was significantly greater than the other two groups. NSA was generally high in these non-ambulatory CP children and was not related to hip displacement and posture.
*Conclusion:* The results did not support a relationship between femoral deformities and hip displacement. Coxa valga and excessive femoral anteversion were general features in non-ambulatory children, no matter hip displacement or not. More anteversion change was noted in the adduction side of windblown posture.

## IEP4

### The treatment of Isolated radial head dislocation in children without annular ligament reconstruction

**Hsuan-Yu Chen**[1], Kuan-Wen Wu[1], Ken N. Kuo[1], Ting-Ming Wang[1]

[1]NTUH, Taipei, Taiwan

**Trauma/Upper extremity**

*Background:* There were number of techniques described in the treatment of neglected isolated radial head dislocation in children. The necessity of annular ligament reconstruction is a controversial issue. The purpose of this study is to report our clinical experience in the treatment of neglected isolated radial head dislocation in children without annular ligament reconstruction.
*Methods:* This is a retrospective review of 10 patients treated for neglected isolated radial head dislocation with ulnar bowing sign between 2008 and 2012. The mean age at surgery was 6 years and 7 months old. The average follow-up period was 15 months. The procedure included open reduction of the radial head and ulnar lengthening angulation osteotomy. There was no need for additional stabilization in cases of stable radial head reduction after open reduction and ulnar corrective-lengthening osteotomy. However, in cases with an unstable radial head reduction, a trans-capitellar K-wire was applied.
*Results:* All 10 patients except one had improved elbow motion clinically. The range of motion of the elbow improved to 120.5° after surgery from 88.5° before surgery. Average pronation-supination arc was 82.5–80° before surgery and 74–81° after surgery. Radiographically, the reduction of radial head was maintained in all cases at the last follow-up.
*Conclusions:* The management of neglected isolated radial head dislocation in children can be difficult. We herein present our surgical strategy to reduce the radial head without annular ligament reconstruction while maintaining reduction with improved functional results.

## IEP5

### Westin tenodesis in calcaneous foot—long-term outcomes

**Patricia Fucs**[1], Helder Yamada[1]

[1]Santa Casa Medical School, Sao Paulo, Brazil

**Hip/lower extremity/foot and ankle**

Westin tenodesis in calcaneous foot—long-term outcomes
Helder H. Yamada, Patricia M. de Moraes Barros Fucs
*Introduction:* Calcaneus foot is present in almost 20 % of the foot deformities in Spina Bifida patients. It is progressive and causes severe gait dysfunction. Tendon transfers are indicate but lack to address the ankle deformity. Westin tenodesis has the potential to correct and stabilize the ankle joint but there is always a fear of overcorrection if it is done in early ages. Long-term outcomes could verify the correlation between the correction and the possible overcorrection of the calcaneus deformity with the Westin technique.
*Patients and methods:* Between 1993 and 2014, 18 spina bifida patients (30 feet) with paralytic calcaneus valgus feet were submitted to the Westin procedure and 15 patients (24 feet) were re-evaluated. Eight patients were male and seven were female. The mean age at the surgical procedure was 7 + 2 (min 3 + 5, max 10 + 10). Surgical indications were: correction of the calcaneous deformity and ankle valgus, decrease the retropulsion during gait and avoid pressure sores. Radiographically measurements made on weight–bearing as follows: on lateral ankle and foot view the calcaneal-tibial (CT) angle and on ap view the intermalleolar height difference and valgus of the ankle joint. Westin tenodesis was done isolated and associated to other procedures. The mean follow-up period was 9 + 10 (min 1 + 2, max 21 + 6). Results were considered satisfactory as improve or correction of the deformities, decrease of the retropulsion and improvement of the gait pattern, increase of the CT angle and decrease of the ankle valgus by lowering intermalleolar difference.
*Results:* Radiographically: the lateral CT angle showed a tendency towards improvement from pre to postoperative. No correlation was found to indicate overcorrection in time, and with the age at surgical procedure. On the other side statistical difference on the correction of the ankle valgus and the intermalleolar difference present. As an overall: 11 (18 feet) patients (74 %) were satisfactory with plantigrade feet. 4 patients (6 feet) unsatisfactory being 2 patients (3 feet) (13 %) no skin problems till the last evaluation and 2 patients (3 feet) (13 %) with pressure sores.
*Conclusion:* No correlation between the follow-up period and the increase of the CT angle, as antecipated. Unsatisfactory results due to pressure sores should be expected in SB patients, especially as they reach adult years.

Keywords: calcaneus foot; calcaneal-fibular tenodesis, Westin technique; Spina Bifida

## IEP6

### Objective gait assessment, perception of gait evolution and quality of life in young adults with cerebral palsy

**Alice Bonnefoy-Mazure**[1], Katia Turcot[2], Yoshi Sagawa[3], Lara Allet[1], Oierre Lascombes[1], Geraldo de COULON[1], Stéphane Armand[1]

[1]University Hospital geneva, Geneva, Switzerland.
[2]University Hospital geneva, geneva, Switzerland.
[3]CHU Besancon, Besancon, France

**Cerebral palsy**

*Background:* The gait evolution of adults with cerebral palsy (CP) on the long term is an important source of information in term of quality of life (QOL), perception and gait assessment.
*Aim* Describe the 10-year gait evolution in young ambulant adults with CP and to investigate its association with their perception of gait evolution and their QOL.
*Method:* Thirteen young adult patients (mean age: 12.6 ± 3.9 years at the first visit, 22.4 ± 5.4 years at the second visit) with a gross motor function classification system (GMFCS) rating between 1 and 3 were included in this study. Two clinical gait analyses were performed, with an interval of 10.5 ± 2.1 years, and the results were used to calculate the Gait Deviation Index (GDI) evolution. A subjective self-evaluation of gait evolution and QOL was administered at the second visit using a visual analogue scale and the Short Form 36 (physical score and mental score) questionnaire, respectively.
*Results:* No significant correlation was found between the evolution of the GDI and the gait scores and the SF-36 scores. However, some patients had good perception of their gait evolution (relative to the GDI), while other patients perceived their gait evolution as being better than that indicated by the GDI.
*Conclusion:* For all patients, the perception of gait improvement was linked to surgical treatment during childhood.

## IEP7

### Patient-reported outcome measures following surgery for developmental dysplasia of the hip

**Paul Cowling**[1], Joshua Craig[1], Rebecca Tate[1], Amar Rangan[1], Richard Montgomery[1]

[1]James Cook University Hospital, Middlesbrough, United Kingdom

**DDH**

*Background:* Outcomes following operative treatment for Developmental Dysplasia of the Hip (DDH) have previously reported variable radiological and functional results. The endpoint of treatment is often total hip arthroplasty. In the UK, Oxford Hip Score (OHS) and Euroqol (EQ-5D-3L) are currently used in adults to demonstrate requirement for Total Hip Arthroplasty. We aim to report an exploratory study investigating investigate the feasibility of using these scores as patient reported outcome measures (PROMs) following surgery for developmental dysplasia of the hip (DDH) in an adolescent population. The oxford hip score (OHS) and Euroqol (EQ-5D-3L) are currently used in adults to assess Total Hip Arthroplasty need and outcome.
*Methods:* We reviewed 33 consecutive patients (36 hips) at a mean of 9.0 years following various forms of DDH surgery (mean age at surgery 2.34 years, range 0.5–9 years). All patients completed the OHS and EQ5D-3L to assess PROMs.
*Results:* Excellent post-operative OHS were reported (mean 47.0/48, range 39–48). EQ-5D-3L was in the top level for each section. Visual analogue health state scale demonstrated a mean of 96.2/100 (range 80–100). These scores were comparable to a normal adult population.
*Conclusions:* This is the first study to review the usefulness of these PROMs following DDH surgery. We found that patients this adolescent population were able to reliably complete PROMs questionnaires, confirming feasibility of the use of PROMs in this age group. However, there is a potential ceiling effect with these the questionnaires used in this study that needs further investigation.

## IEP8

### Treatment of clasped thumb in arthrogryposis

**Hisham Abdel-Ghani**[1], Mostafa Mahmoud[1]

[1]Cairo University, Cairo, Egypt

**Trauma/upper extremity**

The aim of this study is to assess the results and value of treatment of complex congenital clasped thumb in arthrogryposis using modified dorsal rotation advancement flap (Abdel-Ghani H, 2006).
*Patients and methods:* A prospective study on 69 complex clasped thumbs in 39 arthrogrypotic patients underwent surgical reconstruction in the form of release of the web space, skin augmentation using modified dorsal rotation advancement flap and chondrodesis of the metacarpophalangeal joint (MPJ). The patients were assessed regarding parents' satisfaction, thumb position, stability of MPJ and degree of opposition. Follow up ranged from 2 to 7 years (average 3 years).
*Results:* We had non-union of chondrodesis in 7 hands, necrosis of tip of the flap in 7 hands (did not affect the final outcome). No reported long term effect on longitudinal growth of the thumb.
*Outcome of treatment;*

- All parents were satisfied with outcome.
- Thumb position and function: improved ($p < 0.05$).
  - Abduction: 40 excellent, 25 good, 4 fair.
  - Rotation: 30 excellent, 35 good, 4 fair.
  - Opposition: 20 excellent, 19 good, 15 fair, 15 poor.
- MPJ stability: stability achieved in 62 hands ($p < 0.05$)
  - Non-union of chondrodesis in 7 hands.

Two hands with fair stability.
    Five hands with poor instability that needed revision.

*Conclusion*

- No universal criteria for evaluation of results of treatment.
- Using a single criteria (MP extension or web space width) is not enough for assessment of this complex deformity. Global assessment of thumb function and appearance is mandatory.
- Surgical reconstruction improves the cosmetic appearance and function.
- Variable functional outcome is due to variable degree of contractures and muscle involvement.
- Chondrodesis is a good option because of the presence of;

Global instability.
  Abnormal joints.
  Inefficient muscles for transfer.

- Modified dorsal rotation flap is a good option because;

Provide wide rounded web.
  Proximal retraction did not affect the final outcome.
  Easy simple procedure.
  Minimal donor site morbidity.

## IEP9

## Congenital pseudarthrosis tibia

**Gamal Hosny**[1]

[1]Benha Faculty of Medicine, Cairo, Egypt

### Limb reconstructions

Congenital Pseudoarthrosis of the tibia is one of those rare conditions in which no individual surgeon is likely to accumulate enough material on which to base a statistically valid analysis (Nicoll, 1969).We report the management of these cases with bone transport and highlighting the complications.
*Methods:* This sereis incudes 31 cases with an age range from 3 to 16.5 years. The patients were classified according to Crawford classification. The site of the disease at operation was the distal tibia in all cases. The site of the lesion changes due to multiple previous operations. 19 cases had previous operations [1–13].Neurofibromatosis was evident in 17 cases. The operation included application of the circular frame above and below the corticotomy site and then corticotomy was performed followed by debridement of the lesion and application of the rest of the frame which was extended to the foot. Leg length discrepancy ranged from 1 to 17 cm with an average of 7.5 cm. knee motion was normal in all cases. Ankle stiffness from previous treatment was evident in 18 cases. The patients were assessed clinically and radiographically.
*Results:* After a Follow up from 3 to 17 years all cases had primary union except 3. However at last follow up there was nonunion in 7 cases. The limb length inequality was between 0–1 cm in 14,between 1–3 in 13 and more than 3 cm in 4 cases.
*Complications included:* 1-All cases had axial deviations at last follow up except 4. Progressive C shaped valgus osteotomy in 5 cases which had been treated by two osteotomies located away from the site of the original lesion in 4 of them 2-Refractures in 19 cases [1–4 times] 3-Fracture of the regenerate in 3 cases.
*Conclusions:* Ilizarov method has an important role in treatment of congenital pseudarthrosis tibia. However, it is fraught with the problem of residual malalignment in the majority of cases after successful treatment. Besides, the problem of refracture has not been solved yet.

## IEP10

## Characteristic factors that influence the malalignment of lower limbs and can be predictable factors for active management in hereditary multiple exostoses

**Sung Taek Jung**[1], Yeong Seub Ahn[1], Chang Seon Oh[1]

[1]Chonnam Natl. Univ. Hosp., Gwangju, Korea, Republic Of

### Hip/Lower extremity/foot and ankle

*Backgrounds:* The purpose of this study was to describe characteristic factors of the malalignment in hereditary multiple exostoses (HME) involving the lower limbs and identify factors which affect its severity more predictably and demand active treatment.
*Materials and methods:* 32 patients (19 males and 13 females) diagnosed with HME from 2001 to 2013 were evaluated. The mean age at the investigation was 15.0 years (range, 5.0–31.0 years) and the mean follow-up duration was 4.5 years (range, 0–15.5 years). The patients were classified by three methods, based on, location of exostoses, presence or absence of synostosis, and age with growth phase or not. For radiological assessment, mechanical axis (MA) angulation, medial proximal tibia angle (MPTA), lateral distal tibia angle (LDTA), and fibular shortening were evaluated. Longitudinal studies were performed on 20 out of 64 limbs to estimate the severity of the malalignment with the age progression.
*Results:* MA of the lower extremities in the HME seemed to have valgus deformity. High tendency of developing valgus deformity in lower limbs was shown in patients who had proximal or distal tibiofibular joint involvement as observed from MPTA and LDTA measurements. Moreover, the tendency of developing valgus deformity at the ankle as measured by the LDTA was most remarkable ($p < 0.001$). Maximum number of limbs which belonged to the stations higher than or equal to II as per Malhotra classification were most in both proximal and distal joint involvement group ($p < 0.05$). On longitudinal analysis, malalignment of the lower limbs seemed to progress with age, and more so when both the proximal and distal tibiofibular joints were involved.
*Conclusions:* Malalignment of the lower limbs in patients with HME was most severe when both proximal and distal tibiofibular joints were involved and it worsened with the age progression. Therefore, the patients with involvement of both the proximal and distal tibiofibular joints and in growing phase should be considered for more active treatment.

## IEP11

## Hip preservation surgery for post-SCFE osteonecrosis with partial epiphyseal collapse

**Mi Hyun song**[1], Tae-joon Cho[2], Won Joon Yoo[2], In Ho Choi[2]

[1]Jeju National Univ. Hospital, Jeju, Korea, Republic Of.
[2]Seoul National Univ. Hospital, Seoul, Korea, Republic Of

### Hip/Lower extremity/foot and ankle

*Background:* Slipped capital femoral epiphysis (SCFE) is one of the most common causes of osteonecrosis (ON) of the femoral head in children. Literature is scarce regarding the treatment guidelines and outcomes in the management of this condition.
*Purpose:* The purpose of this study was to determine the effectiveness of the flexion intertrochanteric osteotomy with/without coronal and rotational components (FITO) and transtrochanteric rotational osteotomy (TRO) according to the authors' algorithmic treatment strategy.
*Patients and Methods:* Between 2002 and 2012, six patients who presented with post-SCFE osteonecrosis with partial epiphyseal collapse were treated with FITO, and three patients, with TRO. Intra-articular and extra-articular procedures were combined, if needed. The average age at the time of onset of SCFE was 11.7 years (range, 7.8–15.0). Partial ON was diagnosed between 4 and 9 months after initial management. The mean age at the time of the reconstructive operation 13.1 years (range, 9.5–15.5), and the average interval between onset and the reconstructive operation was 17.8 months (range, 6–33). The follow-up period averaged 7.8 years (range, 31–130 months). Remodeling of the femoral head and neck was evaluated on AP and lateral radiographs; alpha angle and beta angle, acetabular-head index, deformity index, sphericity index, and Stulberg grading. In addition, Harris hip score was measured to determine the hip function. All radiological and clinical parameters were compared statistically between pre-operation and post-operation at final follow-up.
*Results:* No patient developed further collapse of the epiphysis after reconstructive operations. Postoperative serial radiographs suggested that redirected hinging segment of the femoral head was remodeled with time to some extent, which contributed to improvement in overall congruency of the hip. The alpha angle and beta angle were postoperatively reduced on average by 52.5o (range, 40.7o to 63.6o) and 37.6o (range, 28.5o to 44.6o), respectively. The average preoperative Harris hip score (HHS) was 63.9 points (range, 42–78), and the average postoperative HHS was 89.6 points (range, 76–97) in those with mid-term follow-ups. However, 3 patients had mild positive impingement signs at last follow-up.
*Conclusions:* Both FITO and TRO appear to be a viable option for treating the hips with ON associated with SCFE in terms of improving congruity, increasing coverage, and promoting containment of the hip.
*Significance:* A well-performed hip joint-preserving operation would be expected to slow the progression of secondary osteoarthritis subsequent to ON after SCFE.

## IEP12

## Aneurysmal bone cysts—is an adjuvant therapy necessary?

**Jirí Chomiak**[1], Pavel Dungl[1], Martin Oštádal[1], Monika Frydrychová[1]

[1]Orthop. Dept. 1st Med Faculty, Prague, Czech Republic

**Tumours/Metabolic**

*Purpose of the study:* Results of different methods of treatment of aneurysmal bone cysts in children age were evaluated.

1. 24 patients (13 boys, 11 girls, age 4–16 years) were treated for aneurysmal bone cysts in period 2001–2012. Most of cysts were primary (19 cysts) and 5 were secondary (two in giant cell tumours, one in fibrous dysplasia, one in non-ossifying fibroma and one in juvenile bone cyst). Locations were mainly in proximal tibia (6 cases), proximal humerus (5 cases) and proximal femur (4 cases). Concerning the treatment modalities, curettage and bone grafting were used 16 patients, curettage + electro-cauterization +bone grafting were used in 2 patients, curettage and bone cement was used in 5 patients and en bloc resection and fibula graft in one patient, respectively. Segment stabilizations were used in 5 patients.
2. 18 cysts healed (Capana grade 1 and 2) after primary procedure, whereas 6 cysts recurred (25 %). The recurrences were 3times in secondary cysts namely in fibrous dysplasia, non- ossifying fibroma and juvenile bone cyst, whereas 3 were in primary cysts. From these recurrences, 5 were treated primarily using the curettage and bone grafting without adjuvant therapy and they represent 31 % od recurrence rate. One recurrence was noted in the group of adjuvant therapy (7 patients), namely after using of bone cement. The recurrence rate represents 14 %. All cysts healed after repeated procedures. There were two serious complications, namely one fracture after bone cement removal and one deep infection. Finally, both patients healed without late sequels for extremity function.

*Conclusion:* Because of unexpected aggressiveness of aneurysmal bone cysts, treatment should be always based on histological evaluation. Curettage and bone grafting with/without periosteal stripping prone to the highest risk of recurrences. Therefore an adjuvant therapy like electro-cauterisation, high speed burr or temporary bone cementation should be preferred. En bloc resection has the lowest recurrence rate but it is useful only in some anatomical locations.

## IEP13

## Comparative analysis of correction rates for titanium-alloy and cobalt-chrome-alloy rods in adolescent idiopathic scoliosis (king type 1 and 2)

**Jae Hyuk Yang**[1], Seung Woo Suh[1], Jin Ho Hwang[2]

[1]Korea Univ, Guro hospital, Seoul, Republic Of Korea.
[2]Yonsei Univ,Severance hospital, Seoul, Republic Of Korea

**Spine**

*Study design:* Prospective comparative study
*Background study* Though numerous biomechanical studies are available in literature, there is no clinical study that has compared curve correction rates for Titanium (Ti) and Cobalt-chrome-alloy (CCM) rods in adolescent idiopathic scoliosis (AIS).
*Purpose of the study:* The goal of this study was to compare the correction rates of Ti and CCM rod materials in AIS curves.
*Methods:* AIS patients who underwent surgery at our scoliosis research center between 2009 and 2012 were enrolled. Among them patients who met following criteria were included: Risser's stage 1–4, 11–17 years at surgery, rotation more than Nash Moe grade II and King type 1 or 2 curves (double curves) with preoperative Cobb's angle of major curvature more than 50° and secondary curve more than 30°. Correction surgery was performed through posterior approach using rod-rotation maneuver after inserting pedicle screw in every vertebrae within the fusion. Six mm CCM and 6-mm Ti rods were used in experimental and control group respectively. Pre-operative and post-operative indices of coronal alignment (Cobb's angle, coronal balance, T1-tilt, clavicle angle) and sagittal alignment (spinal vertical axis, thoracic kyphosis, lumbar alignment) were measured.

*Results:* Total 45 patient were included (experimental group: 16, control group: 29). There was no statistical difference between two groups for age, sex, Risser stage, pre-operative Cobb's angle, type and flexibility of curvature ($p > 0.05$ for all factors). In experimental group, correction rate for thoracic and thoracolumbar curvature were 71.2 and 66.8 %. In control group, correction rate of thoracic and thoracolumbar curvature were 71.2 and 73.3 % ($p = 0.664$ and 0.09). There were no statistical differences between two groups for all coronal and sagittal factors ($p > 0.05$)
*Conclusion* This study concludes that for same rod diameter (6 mm), Ti and CCM rods had similar coronal and sagittal correction rates in AIS.

Keywords: Spine; Scoliosis; Cobalt-chrome; Titanium; Correction rate:

## IEP14

## Proteomic analysis of the extracellular matrix in clubfoot

**Martin Ostadal**[1], Adam Eckhardt[2], Jiri Chomiak[1], Monika Frydrychová[1], Pavel Dungl[1]

[1]University Hospital Bulovka, Prague, Czech Republic.
[2]Academy of Sciences, Prague, Czech Republic

**Club-foot**

*Background:* Idiopathic *pes equinovarus* is a congenital deformity of the foot and lower leg defined as a fixation of the foot in adduction, supination and varus. Although the pathogenesis of clubfoot remains unclear, it has been suggested that fibroblasts and growth factors are involved.
*Objective:* To directly analyze the protein composition of the extracellular matrix in contracted tissue of patients with clubfoot.
*Methods* A total of 13 infants with idiopathic clubfoot treated with the Ponseti method were included in the present study. Tissue samples were obtained from patients undergoing surgery for relapsed clubfeet. Contracted tissues were obtained from the medial aspect of the talonavicular joint. Protein was extracted after digestion and delipidation using zip-tip C18. Individual collagenous fractions were detected using a chemiluminiscent assay.
*Results:* Amino acid analysis of tissue samples revealed a predominance of collagens, namely collagen types I, III and VI. The high content of glycine and h-proline suggests a predominance of collagens I and III. A total of 19 extracellular matrix proteins were identified. The major result of the present study was the observation that the extracellular matrix in clubfoot is composed of an additional 16 proteins, including collagens V, VI and XII, as well as the previously described collagen types I and III and transforming growth factor β.
*Clinical relevance:* The characterization of the general protein composition of the extracellular matrix in various regions of clubfoot may help in understanding the pathogenesis of this anomaly and, thus, contribute to the development of more efficacious therapeutic approaches.

Open Access

This article is distributed under the terms of the Creative Commons Attribution License which permits any use, distribution, and reproduction in any medium, provided the original author(s) and the source are credited.

